# Efficacy of mind maps and concept maps in enhancing academic performance among undergraduate medical students in the preclinical stage: a systematic review

**DOI:** 10.1007/s10459-025-10437-4

**Published:** 2025-06-24

**Authors:** Husam Aljamal, Rama Alawneh, Afnan Derbas, Mohammad Edaibes, Aya Ahmed, Lama Amer, Hiba Alzoubi, Hashem Abu Serhan

**Affiliations:** 1 Faculty of Medicine, Yarmouk University, Irbid, Jordan; 2 Department of Ophthalmology, Hamad Medical Corporation, Doha, Qatar

**Keywords:** Mind maps, Concept maps, Academic performance, Medical students, Pre-clinical education

## Abstract

**Purpose:**

This systematic review aims to evaluate the impact of mind maps and Concept Maps on academic performance among undergraduate medical students in the preclinical stage.

**Methods:**

Our protocol was registered on PROSPERO (CRD42024529458). We followed PRISMA guidelines in conducting our systematic review. We searched PubMed, Scopus, Google Scholar, and Cochrane Library from inception to June 2024. We included only randomized controlled trials (RCTs) that involved undergraduate medical students in the preclinical stage, evaluated mind mapping and Concept Maps as the intervention, and compared it to traditional methods or no intervention, with academic performance as the primary outcome. A qualitative synthesis of the results was conducted, and where possible, effect sizes (e.g., Cohen’s d) were calculated to quantify the impact of mind maps and concept maps on academic performance. The risk of bias was assessed using the Cochrane Risk of Bias tool. The risk of bias was assessed using the Cochrane Risk of Bias tool.

**Results:**

A total of six RCTs were included. Four studies reported significantly higher assessment scores with the use of mind maps or concept maps. Teli et al. (Indian Journal of Clinical Anatomy and Physiology *7*(2):243–246, 2020) observed a significant improvement in knowledge retention in the intervention group (mean score: 82.4% vs. 69.8%, *p* < 0.0001). Concept maps demonstrated moderate-to-large effect sizes, with Ho et al. (Medical Education 48(7):687–697, 2014) reporting Cohen’s d = 0.7–0.8 for improved test scores. Two studies found no significant differences in performance (*p* > 0.05), but students consistently expressed a preference for these methods due to their utility in understanding and summarizing information. Overall, mind maps and concept maps prove to be effective tools for enhancing academic performance, especially in terms of knowledge retention and comprehension.

**Conclusion:**

This systematic review shows that mind maps and Concept Maps are effective methods in helping undergraduate preclinical medical students achieve better performance, especially in terms of knowledge retention and comprehension. While not all studies showed significant differences, the overall preference for these methods indicates their potential as valuable learning tools. We recommend integrating these tools into preclinical curricula and providing training sessions to enhance their effectiveness.

**Supplementary Information:**

The online version contains supplementary material available at 10.1007/s10459-025-10437-4.

## Introduction

In today’s world, students apply for medical school for a variety of reasons, which include job satisfaction, the institution’s high prestige in society (de Oliveira Vasconcelos Filho et al., 2016), a strong appreciation of the humanistic aspects of medicine, and an early desire to help people (Millan et al., 2005). Even though many students often mention that a family member influenced their decision (Pruthi et al., 2013). However, many of these students are unaware of the actual challenges they will encounter, which include time management and study issues, problems with relationships and work-life balance, peer connections in medical school, health issues, and financial strains (Hill et al., 2018).

Given that the medical sector is recognized for being extremely knowledge-based, medical students reported in a study conducted across all Florida schools that the highest stressor during their academic careers was the heavy academic workload (Hill et al., 2018). According to a study at the University of São Paulo regarding the difficulties expected during the medical course, a lack of time was reported as the primary issue (Millan et al., 2005). This suggests that students should deal with a vast amount of information in a very short time just for the exam, which has detrimental effects on their study quality.

Due to the rapid change in medical education and its associated quantity of knowledge and complexity, a significant transformation has taken place recently in the approach to medical education. It has become necessary to replace the traditional methods of passive learning through lectures with student-centered, active learning methodologies that utilize a variety of innovative teaching method (Bala et al., 2016; Beigzadeh & Haghani, 2015; Daley & Torre, 2010; Deshatty & Mokashi, 2013; Gutierrez et al., 2016; Ho et al., 2014; Ho & Velan, 2016; McCoy et al., 2018; Mlika et al., 2017; Schwartzstein & Roberts, 2017). One of the major challenges for medical teachers is to share expert medical knowledge and to implement these newer educational methods during teaching and learning activities (Bala et al., 2016; Beigzadeh & Haghani, 2015; Oliveira et al., 2023; Qadir et al., 2011). Numerous studies have demonstrated the efficacy of such active learning techniques in enhancing students’ academic performance (Freeman et al., 2014; Kay & LeSage, 2009; Kim, 2015; Kwan et al., 2017; Langenau et al., 2017; Lindeman et al., 2015). Based on different research, two strategies that can lead to meaningful learning are concept mapping (Ertmer & Nour, 2007; Kinchin et al., 2008; Mayer, 1989; Novak & Gowin, 1984) and mind mapping (Davies, 2011).

Visual learning techniques, such mind maps and concept maps, have been shown in cognitive psychology research to improve information organization and long-term retention (Novak & Gowin, 1984). Research in the natural sciences and engineering has also shown that concept mapping facilitates critical thinking and problem-solving (Kay & LeSage, 2009). Our study highlights the domain-specific applicability of these methods in organizing complicated medical information while confirming their importance by combining ideas from medical education with these more general findings.

The concept map, a schematic device for representing a set of concept meanings embedded in a framework of propositions, was first developed by Joseph Novak and Bob Gowin at Cornell University in 1972 (Novak & Gowin, 1984). It was based on Ausubel’s theory that learning occurs by the assimilation of new concepts and propositions into existing concepts and propositional frameworks held by the learner (Cañas et al., 2003). Mind maps, a graphic tool with words, ideas, and pictures radiating out from a central word or idea, were created by Tony Buzan in the 1970s (Solati, 2015). Both tools provide a graphic representation of knowledge that can easily be understood by others (Buzan & Buzan, 2010; Novak, 2010). These maps have been widely used in educational activities with different purposes, e.g., in medical education (D’Antoni et al., 2009, 2010; Daley & Torre, 2010). So, both tools are included in this review because they share the common goal of enhancing knowledge organization and retention, despite their structural differences.

However, two main characteristics differentiate concept maps from mind maps: (Santiago, 2011) (a) Hierarchical: Concept maps are hierarchical with the most important concept shown first, usually at the top of the map. Subsidiary concepts are placed below the main concept. Tertiary concepts derived from secondary concepts are placed below secondary ones. This process continues as needed. In mind mapping, the main idea is placed at the center of the map, and all other ideas are outgrowths of the main idea with no obvious hierarchy. (b) Explicit naming of links: Concept mapping requires that the links between concepts are named explicitly through verbs such as “includes,” “is part of,” etc. Naming the links allows for an easier and more accurate interpretation of the map. Mind maps do not name the links, and the nature of the relationship is implicit.

The benefits of using these learning and teaching strategies are numerous. Their expanded use in pre-clinical courses may help students explore multiple hypotheses before deciding on a diagnosis and reduce cognitive errors and misdiagnosis (Ramos, 2015; Veronese et al., 2013). The use of pictures and colors, together with words in these maps, enables students to combine two different cortical skills that enhance intellectual power and facilitate the conversion of information from short- to long-term memory (Day & Bellezza, 1983), probably because the brain processes images a lot faster than text (Ramos, 2015). It also aids in studying, organizing, summarizing information, and writing. It is an effective method of taking notes and aids in the recall of existing memories (Deshatty & Mokashi, 2013). Moreover, in medical education, different research suggests that concept and mind maps can be an effective tool to bridge the gap between basic and clinical practice by allowing students to link new knowledge with existing one (Beigzadeh & Haghani, 2015; Oliveira et al., 2023; Spicer et al., 2017; Veronese et al., 2013). However, making insightful and thorough maps takes a lot of time, adding to the already demanding academic burden placed on students. Students often require multiple practice sessions before they become proficient in developing useful concept maps (Liu et al., 2023). This difficulty is particularly noticeable in industries like medicine, where students find it impossible to develop mind maps for every topic due to the vast number of subjects covered (Veronese et al., 2013).

While both concept maps and mind maps have been extensively employed in educational contexts, little is known about the precise effect they have on academic achievement, especially for preclinical undergraduate medical students. Despite plenty of research on active learning techniques. Few systematic reviews have focused on the comparative effectiveness of mind maps and concept maps in medical education. This systematic review is the first to focus specifically on the efficacy of mind maps and concept maps in enhancing academic performance in this group and offers a high degree of evidence by synthesizing findings from randomized controlled trials (RCTs) to offer a unique comparative perspective. By analyzing both tools together. We provide useful insights for educators looking to maximize learning processes in preclinical medical education by incorporating both tools…

## Methods

### Protocol and registration

This systematic review was conducted according to a pre-established protocol that was registered with PROSPERO (CRD42024529458) on March 27, 2024. The methodology adhered to the guidelines set by the Preferred Reporting Items for Systematic Review and Meta-Analysis (PRISMA) (Moher et al., 2009). There were some deviations from the original protocol which were edited on July 4, 2024, because we planned to conduct a meta-analysis; however, the quality of the data and the differing methodologies across the six studies made it challenging to perform a meaningful meta-analysis. Instead, we conducted a qualitative synthesis and where possible, effect sizes (e.g., Cohen’s d) were calculated.

### Eligibility criteria

Studies were considered eligible if they involved undergraduate medical students in the preclinical phase, examined the impact of mind mapping or concept mapping as an intervention, and compared it with traditional learning methods or no intervention. The primary outcomes assessed were academic performance metrics such as exam scores and course grades. Only randomized controlled trials (RCTs) published in English between 2000 and 2024 were included. Studies not meeting these criteria were excluded.

We included both mind maps and concept maps in this review because, despite their structural differences, they serve a common purpose: enhancing knowledge organization and retention in medical education. Mind maps encourage associative learning and rapid recall, while concept maps facilitate deeper hierarchical structuring of knowledge. By incorporating both, we were able to present a more comprehensive view of the efficacy of visual learning techniques in preclinical instruction.

### Information sources and search strategy

A systematic search was performed across four major databases: PubMed, Scopus, Google Scholar, and the Cochrane Library. We designed the search strategy to capture all relevant studies published between 2000 and 2024. The search terms used included (“mind map*”) OR (“concept map*”)) AND (“medical student*”), with publication date filters from 2000 to 2024. We used Boolean operators (AND, OR) and truncation symbols (*) to ensure comprehensive search. The last search was conducted in June 2024. Detailed search strategies are available in Supplementary Table 1.

### Study selection

**Fig. 1:**
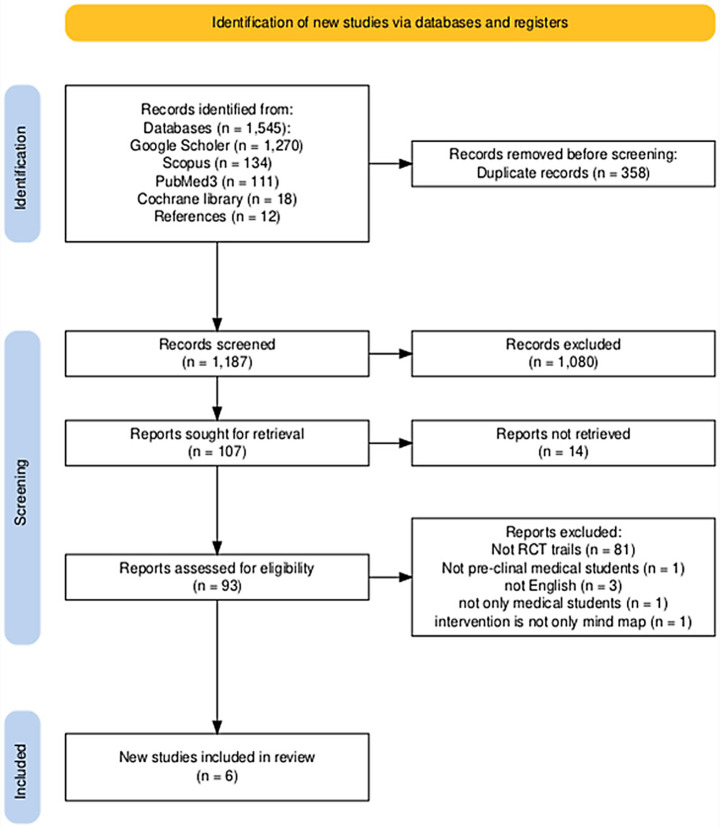
PRISMA Flow Diagram. This diagram illustrates the flow of information through the different phases of the systematic review, including identification, screening, eligibility, and inclusion of studies

Before starting the screening process, records that were retrieved from database searches (PubMed, Scopus, Google Scholar, and Cochrane Library) were exported into Mendeley to eliminate duplicates. Two reviewers then independently screened titles and abstracts based on predefined inclusion criteria. Any differences or doubts were settled by conversation or by seeking advice from a third reviewer. Articles in full text were evaluated for eligibility in the second screening stage to decide whether to include the whole texts, two reviewers separately examined them. Discussions were held to settle any differences, and a third reviewer was consulted to reach a conclusion if no agreement could be achieved. The PRISMA flow diagram (Fig. 1) presents a complete overview of the research selection procedure. We utilized an online tool to create this flow diagram (Haddaway et al., 2022).

### Data extraction

Data extraction was conducted using a standardized form, which was piloted on a subset of studies to ensure consistency. Two reviewers independently extracted key data such as study characteristics (author, year of publication, country), participant demographics (age, sex, year of study), intervention details (type, duration, frequency of mind mapping sessions), comparison groups (traditional learning methods, no intervention), outcomes (exam scores, course grades), and risk of bias assessments using the Cochrane Risk of Bias tool. Any discrepancies were resolved through discussion or by consulting a third reviewer. The extracted data were cross-checked for accuracy, and a summary table (Table 1) was created to present the baseline characteristics of the included studies.

**Table 1:** Baseline characteristics of included studies

Study	Name	Year	Country	Participants	Sample Size	Intervention	Comparasion	Outcome Measures	Duration	Intervention Sample Size	Intervention Mean	Intervention SD	Control Sample Size	Control Mean	Control SD	Aim	Conclustion
1	Surapaneni & Tekian	2013	India	- First-year medical students - From Saveetha Medical College and Hospital in India - Total of 150 participants	Total: 150 - Traditional program: 75 - Innovative program: 75	The intervention was concept mapping with clinical cases, done in small groups of 5 students with 3 units total, following a 6-step process (brainstorming, organizing, layout, linking, revising/finalizing, gallery walk) under the supervision of a faculty member. This intervention was only given to the “innovative group” of 75 students, while the other 75 students received the traditional lecture-based program.	Traditional lecture-based program	1) Student performance on three written knowledge tests, each with a maximum score of 20 2) Student perceptions of the relevance and usefulness of the learning process, measured using a 12-item questionnaire	6 months	75	• Test 1: 12.33 • Test 2: 13.93 • Test 3: 13.39	• Test 1: 3.17 • Test 2: 1.67 • Test 3: 1.51	75	• Test 1: 7.99 • Test 2: 8.28 • Test 3: 7.13	• Test 1: 2.34 • Test 2: 1.40 • Test 3: 1.26	- Design and implement an innovative curriculum consisting of concept mapping with clinical cases - Compare the knowledge of undergraduate medical students in the traditional curriculum vs. the innovative curriculum - Understand the perceptions of undergraduate medical students on the usefulness of concept mapping with clinical cases in learning biochemistry	The use of concept-mapping techniques resulted in significantly better test scores and promoted a deeper understanding of the basic concepts of biochemistry to relate and link to medical problems.
2	Teli et al	2020	Not mentiond	First-year MBBS students	Total: 80 - Mind mapping group: 40 - Didactic lecture group: 40	1) Mind mapping for 45 minutes, followed by clarification of doubts 2) Didactic lecture using a blackboard for 45 minutes, followed by clarification of doubts	Didactic lecture	Knowledge scores of students learning neuroanatomy, and students’ perceptions about mind mapping as a teaching method	110 min	40	• Session I (Spinal Cord): 2.53 • Session II (Thalamus): 2.10	• Session I (Spinal Cord): 1.48 • Session II (Thalamus): 1.16	40	• Session I (Spinal Cord): 3.83 • Session II (Thalamus): 4.18	• Session I (Spinal Cord): 1.12 • Session II (Thalamus): 1.13	- To compare mind mapping and didactic instructional method in learning neuroanatomy for first MBBS students - To assess students’ perception about mind mapping	Students perceived Mind mapping as interesting, innovative and enjoyable method of learning. Mean Knowledge scores of students in mind mapping group were better than students in didactic lecture group. The difference is proved statistically significant by quantitative and qualitative test. We conclude Mind mapping is effective in teaching complex conceptual subjects. Mind mapping, a student-centered teaching learning method because of active participation, analytic thinking and visual presentation helps in better recall.
3	Widanapathirana et al	___	Sri Lanka	- New entrant medical students at the Faculty of Medicine, University of Colombo - Randomly assigned to two equal groups based on university entrance exam performance	74	The intervention was a 30-minute lesson on how to create mind maps, followed by 45 minutes to read the text and create/study a mind map.	Self-Selected Study Technique Group (SSG): Used their existing study methods	Academic performance on structured essay questions	45 mintues	34	31.30%	Not provided	34	37.60%	Not provided	- To evaluate the effectiveness of using mind maps as a self-learning method for new medical students - To introduce the mind map technique to new medical students	The mind map technique did not show any superiority over other conventional study techniques as a short term learning method in a newly trained population. But the majority of the mind map group perceived it as a useful way of summarising information. They also perceived it to be helpful in memorising information in an organized manner compared to their previous self study techniques.
4	Kalyanasundaram et al	2017	India	Sixth semester MBBS students	Total: 64 - Text group: 32 - Mind map group: 32	1) Text reading method: One group of 32 participants received a 1500-word text passage and were asked to read it using the routine text reading method for 20 minutes. 2) Mind mapping technique: The other group of 32 participants received a 20-minute session where they were taught the mind mapping technique, and then they were asked to use the mind mapping technique to learn the same passage received by the other group.	Routine text reading method	Knowledge gain (change in mean score between baseline and day 0, and between baseline and day 7)	20 minutes	32	3.5 (baseline), 9.0 (Day 0), 8.9 (Day 7)	Not provided	32	2.6 (baseline), 8.7 (Day 0), 8.5 (Day 7)	Not provided	To assess the impact of mind mapping technique in information retrieval among medical college students in Puducherry.	The mean score immediately after intervention is slightly higher in the mind map group than the text group, the difference is statistically not significant.
5	Ho et al	2014	Australia	medical students in Years 1 and2 enrolled in Phase 1 of a 6-year undergraduate medicine programme	65	Access to testable online pathogenesis maps relating to key concepts in pathology covered in each 2-week block (renal, hepatic, and pancreatic disease), or links to existing online resources (Robbins Basic Pathology, 9th edition online via MD Consult) relating to the same concepts, in a crossover design	Existing online resources	Performance on online quizzes, including questions related to the topics covered in the pathogenesis maps and unrelated questions	2 weeks	33	Not provided	Not provided	32	Not provided	Not provided	- To evaluate the quantitative impact of online testable pathogenesis maps on the learning of pathology by medical students, compared to traditional learning methods - To evaluate the qualitative impact (acceptability) of online testable pathogenesis maps on the learning of pathology by medical students	The results of this randomised crossover study indicate that online, testable, scaffolded concept maps are well accepted by junior medical students and can improve their learning of concepts in pathology
6	Mukhopadhyay et al	2019	Kolkata, India	− 5th semester MBBS students at ESI-PGIMSR & ESIC Medical College, Joka, Kolkata - Willing to participate and gave written informed consent	75 (48 analysied - Concept map group: 22 - Tutorial group: 26)	The interventions were: 1) Concept map method: Groups A and B were taught using the concept map method after an initial didactic lecture. 2) Traditional tutorial method: Groups C and D were taught using the traditional tutorial method after the initial didactic lecture.	Traditional tutorial methods	Student performance on a questionnaire assessing knowledge of drugs affecting calcium metabolism	2–3 months	22	Not provided	14.81	26	6.76	9.87	- To study the effectiveness of concept map as a reinforcement tool in medical teaching, in comparison to the usual tutorial method - To find the students’ perception towards the concept map teaching method	Students found that concept maps enhanced their capacity to get a comprehensive and accurate overview of the entire topic. It is an excellent choice as a reinforcement tool reflecting the performance score but has not shown better performance than traditional tutorial (didactic lecture) method statistically.

### Quality assessment

The risk of bias in included studies was assessed using the Cochrane Risk of Bias tool (Higgins et al., 2011). Two reviewers independently evaluated each study for potential biases in randomization, allocation concealment, blinding of participants and personnel, blinding of outcome assessment, incomplete outcome data, selective reporting, and other sources of bias. Discrepancies were resolved through discussion. The results of the risk of bias assessment are presented in Supplementary Figures 1 and 2, which provide a visual representation of the risk of bias across studies. Studies were categorized as having low, moderate, or high risk of bias based on these assessments.

### Data items

Extracted data included study characteristics (author, year of publication, country), participant demographics if mentioned (age, sex, year of study), intervention details (type, duration, frequency of mind mapping sessions), comparison groups (traditional learning methods, no intervention), outcomes (exam scores, course grades), and risk of bias. Discrepancies were resolved through discussion.

### Summary measures

The primary measure was the mean difference in academic performance (exam scores and course grades) between the intervention and control groups. Secondary outcomes included participant satisfaction and retention rates.

### Synthesis of results

We could not perform a meta-analysis due to the heterogeneity among the included studies. These differences included outcome measurement (e.g., multiple-choice tests vs. essay-based assessments), intervention design (e.g., single-session vs. multi-week programs), and statistical reporting (e.g., some studies reported means and standard deviations, while others did not). Instead, we conducted a structured narrative synthesis and presented the results as narrative summaries that highlight key findings and their implications. We reported effect sizes (e.g., Cohen’s d) to provide a quantitative comparison of outcomes. Additionally, we performed a thematic analysis to identify common patterns across studies, further strengthening the synthesis. Multiple reviewers independently coded the data and compared results to ensure consistency and reduce bias.

### Risk of bias across studies

We assessed Publication bias and selective reporting qualitatively due to the limited number of included studies.

## Results

The objective of this systematic review was to evaluate the efficacy of mind maps in enhancing academic performance among undergraduate medical students in the preclinical stage. The initial database search yielded 1,545 studies. After removing duplicates, 1,187 studies were screened based on titles and abstracts. Subsequently, 97 full-text articles were reviewed for eligibility, and six RCTs met the inclusion criteria (Ho et al., 2014; Kalyanasundaram et al., 2017; Mukhopadhyay et al., 2019; Surapaneni & Tekian, 2013; Teli et al., 2020; Wickramasinghe et al., 2007). These studies were included in the final analysis, with the study selection process detailed in the PRISMA flow diagram (Fig. 1).

### Quality assessment

We used the Cochrane risk of bias assessment tool and found a low to moderate risk of bias among the included studies. All six studies adequately reported their methods of randomizing participants, but allocation concealment was of an unclear risk in two studies (Kalyanasundaram et al., 2017; Surapaneni & Tekian, 2013), a high risk in two studies (Mukhopadhyay et al., 2019; Wickramasinghe et al., 2007) and low risk in two other studies (Ho et al., 2014; Teli et al., 2020). Blinding of participants and personnel was not applicable in all studies due to the nature of the intervention, blinding of outcome assessment was reported in two studies (Kalyanasundaram et al., 2017; Mukhopadhyay et al., 2019), the risk was unclear in three studies (Ho et al., 2014; Surapaneni & Tekian, 2013; Wickramasinghe et al., 2007), and the risk was high in only one study (Teli et al., 2020). Incomplete outcome data were adequately addressed in four studies (Ho et al., 2014; Kalyanasundaram et al., 2017; Surapaneni & Tekian, 2013; Teli et al., 2020) and the risk was high in two studies (Mukhopadhyay et al., 2019; Wickramasinghe et al., 2007). All studies had a low risk of selective reporting bias or any other bias (Supplementary Figs. 1 and 2).

### Study characteristics

The characteristics of the studies included are shown in Table 1 which gives a detailed overview including sample size, type of intervention, groups of comparison, outcome measures, duration, aim, and conclusion. Specifically, the studies concerned medical students from different countries: India (Kalyanasundaram et al., 2017; Surapaneni & Tekian, 2013), Sri Lanka (Wickramasinghe et al., 2007), and Australia (Ho et al., 2014). The educational levels of the participants ranged from first year to third-year MBBS students. The total number of participants across all studies was 503 and the sample sizes were from 64 to 150 participants, The gender distribution was specified only in the study by Kalyanasundaram et al. (2017), which included 30 males (46.9%) and 34 females (53.1%). Age details were not specified in any of the studies. The methods were traditional lecture-based programs, mind mapping, concept mapping, and online resources. The outcome measures evaluated included the student’s performance on knowledge tests, perceptions of learning methods, and academic performance on structured essay questions. The duration of the intervention was from a single 45-minute session to six months. The studies had diverse aims, focusing on the evaluation of innovative teaching methods, such as concept mapping and mind mapping, in medical education improvement. Overall, the studies found that interactive and visual learning techniques were well-received by students and had the potential to enhance academic performance and understanding of medical concepts (Table 1).

### Synthesis of results

The systematic review of six RCTs thoroughly evaluates the effectiveness of mind maps and concept maps in boosting academic performance among undergraduate medical students in the preclinical stage. The majority of studies indicate that these tools significantly enhance knowledge retention, recall, and assessment performance compared to traditional study methods.

Among the studies evaluating mind maps, Teli et al. (2020) and Kalyanasundaram et al. (2017) both found that mind maps significantly improved knowledge retention and recall. Teli et al. (2020) noted that the intervention group scored much higher on assessments (*p* < 0.0001), while Kalyanasundaram et al. (2017) observed better scores immediately after the intervention (mean score: 7.2 vs. 6.5, *p* = 0.047) and one week later (mean score: 8.1 vs. 7.1, *p* = 0.012).

Surapaneni & Tekian (2013) and Ho et al., (2014) demonstrated that using concept maps, whether integrated with clinical cases or as online testable maps, led to improved test performance. Surapaneni & Tekian (2013) reported mean scores of 12.33 to 13.93 in the intervention group versus 7.13 to 8.28 in the control group (*p* < 0.001). Ho et al. (2014) found significant score improvements in quizzes on renal (mean score: 56.0% vs. 46.1%, *p* < 0.01, Cohen’s d = 0.8) and hepatic diseases (mean score: 66.1% vs. 56.8%, *p* < 0.01, Cohen’s d = 0.7), suggesting large effect sizes for concept mapping in structured medical learning. On the other hand Mukhopadhyay et al. (2019) and Wickramasinghe et al. (2007) reported no significant differences in performance between the mind map or concept map groups and their respective control groups. Mukhopadhyay et al. (2019) found no significant difference in last semester’s scores (*p* = 0.9866) or post-test scores (*p* = 0.8682). Both groups, however, showed significant within-group improvements from pre-test to post-test scores (concept map group: *p* = 0.0046, tutorial group: *p* = 0.0078), indicating some learning benefits despite a lack of between-group significance. Similarly, Wickramasinghe et al. (2007) observed no significant performance difference between the mind map group (31.3%) and the control group (37.6%). Despite this, 97.1% of students in the mind map group found the method helpful for summarizing information and expressed a strong interest in continuing its use. Additionally, over 90% of students in Mukhopadhyay et al.’s (2019) concept map group favored this method for a better understanding of the topic.

Across all studies, structured interventions using mind maps and concept maps resulted in mean score increases ranging from 5% to 15%, suggesting a moderate learning benefit. In courses that required a deep integration of concepts (such as pathophysiology), concept maps were more successful for structured learning and knowledge application, but students more often favored mind maps for information organization and comprehension improvement.

In summary, these RCTs suggest that mind maps and concept maps are effective tools for enhancing academic performance, particularly in knowledge retention and comprehension. Although they may not always lead to significantly higher raw scores compared to traditional methods, their perceived utility and acceptance among students highlight their value in medical education.

## Discussion

This systematic review demonstrates that mind maps have the potential to become a useful learning tool in preclinical medical education. Our main finding is that mind maps can enhance academic performance by improving knowledge retention and understanding. Four out of six studies (Ho et al., 2014; Kalyanasundaram et al., 2017; Surapaneni & Tekian, 2013; Teli et al., 2020) demonstrated statistically significant improvements in academic results with the use of mind maps, while the remaining two studies (Abdulghani et al., 2014; Mukhopadhyay et al., 2019), although not showing significant differences, still indicated a preference for mind maps among students. This suggests that mind maps may offer perceived benefits that contribute positively to the learning experience. The study intervention duration, type of assessment, design difference, are reasons for the varied study outcomes. The authors have stated that studies which were able to notice a significant improvement were the ones that have integrated these map types well and continued their implementation within the curriculum gradually. Instead, studies without significant findings (e.g., Teli et al. 2020) often were shorter intervention durations, or the reinforcement strategies were absent. Hence, the way that mind maps and concept maps are incorporated determines their productivity. Suggestions for future research papers may revolve around how instructional static display and relearning will help to influence the outcome of the learning assessment.

Literature supports our findings, with studies by Abdulghani et al. (2014) Farrand et al., (2002), and Day and Bellezza (1983) confirming our findings in bringing forth the concept that mind maps allow students to contextualize and remember tremendous volumes of information to create the big picture which links new information with prior knowledge. This combination of words and visualizations favors better retention of information as noted in Farrand et al.’s (2002) study further which found improved performance among students using mind maps.

Moreover, while Most of the studies are confined to short-run measures of academic performance; some like. Farrand et al. (2002) study evaluated long-term recall showing enhanced recall after one week. That of course makes sense with the cognitive processing advantages we discussed but also highlights an awareness for long-term retention studies since most of our studies looked at short-term gains.

Most of the studies we included focused on preclinical education, without mentioning the use of mind maps in clinical teaching or the development of clinical reasoning. The study by Mollberg et al. (2011) and Kibble et al. (2014) expands this realm by demonstrating that mind maps are useful not only in preclinical settings but also in the organization of clinical information and in facilitating clinical reasoning. Mind maps were used to analyze the clinical reasoning of trainees and to enhance their capability for the holistic management of patients. This extends the utility of mind maps into practical, real-world medical training, extending beyond the scope of the preclinical focus in our review.

Limitations of our review include considerable variations in the design and detail of the interventions; for example, duration of use of mind maps, nature of assessments of academic performance, and discipline in which mind maps were used. This heterogeneity may affect the results and can limit drawing a final conclusion regarding effectiveness across all preclinical medical subjects. Furthermore, several studies reported small sample sizes, and that could be restrictive toward generalizing the results. Another limitation that might be considered is the subjective nature of student preference for mind maps, which may not always correlate with objective academic outcomes. Further research needs to make more precise the link between subjective preferences and objective performance, examining long-term retention rather than immediate test results. Most of the studies measured performance for only brief periods following instruction, with limited analysis regarding the effectiveness of mind maps on long-term retention and understanding. Medical education requires not only mastery at the time of learning but application of knowledge throughout a lifetime career. Research into how mind maps impact long-term retention and application in a clinical environment can yield much-needed lessons about their wider applicability within medical training. Most of the included studies demonstrated a short-term increase in performance, leaving gaps in understanding the long-term effectiveness of the mind and concept maps in knowledge retention and clinical application. While one study (Farrand et al., 2002) discovered that participants could remember more information after a week. These findings have not been consistently confirmed; therefore, more research is required to determine whether these strategies facilitate long-term knowledge retention and clinical decision-making. Furthermore, the authors of the coming works should inquire about the ways that mind-mapping methods will improve critical thinking, problem-solving, and self-managed learning apart from PCE. The new digital learning technology has been swiftly incorporated into medical education, and it is obvious that investigating the function of digital minds and concept maps in promoting a large scale, fast, and comprehensive memory is an important area for future studies. Researchers should search for digital tools in mapping whether they improve the outcome in learning compared with the paper- based method and the ways the tools affect self- directed studies in virtual medical education contexts.

Our results demonstrate that while both concept maps and mind maps enhance academic achievement, their practical applications vary. Concept maps allow knowledge to be organized hierarchically, but mind maps encourage associative learning and quick recall. The strategic placement of mind maps and concept maps is determined by the learning goals. Mind maps are particularly well-suited for summarizing key concepts and promoting rapid recall, making them ideal for early-stage learning and revision. In contrast, concept maps facilitate deep integration of knowledge and are more effective for subjects requiring complex problem-solving and hierarchical reasoning, such as pathophysiology.

To maximize the effectiveness of these tools, educators should provide structured guidance on how to create and use them. It is important for educators to guide students through the process of creating these maps. So, one approach is to incorporate mind maps into weekly review sessions or pre-exam study plans, allowing students to actively consolidate key concepts. Furthermore, adding mapping exercises to tests, for example, asking students to create a concept map rather than a conventional essay question—could encourage active engagement with the subject. Educators should also encourage collaborative mapping, where students build maps in small groups. Teachers can increase the benefits of these strategies for both short-term knowledge retention and long-term clinical reasoning by intentionally including them into their lessons. The long-term effects of these tools on clinical reasoning and retention beyond preclinical instruction should be investigated in future studies, for example multi-year follow-up studies could help determine whether these tools improve diagnostic accuracy and decision-making in real-world settings.

## Conclusion

The findings of this review suggest that preclinical medical students can improve their academic performance by using mind maps and concept maps. These tools not only improve comprehension and knowledge retention but also promote self-directed learning, an essential skill for lifelong medical education. Medical educators should actively include mind maps into their lesson plans, especially for courses that call for the synthesis of complex information. Incorporating these techniques into problem-based learning (PBL), case discussions, and revision sessions could help students to develop stronger critical thinking and organizational skills. Additionally, structured training on how to effectively create and use these maps may further enhance their educational impact. To further establish mind maps’ usefulness in medical education. Future research should focus on the long-term educational benefits of these tools, particularly their role in clinical reasoning, diagnostic accuracy, and decision-making in patient care. Larger, multi-institutional studies with longitudinal follow-ups could provide deeper insights into how these strategies contribute to knowledge retention beyond the preclinical years.

## Electronic supplementary material

Below is the link to the electronic supplementary material.

Supplementary Material 1

## Electronic supplementary material

Below is the link to the electronic supplementary material.

Supplementary Material 2

## Electronic supplementary material

Below is the link to the electronic supplementary material.

Supplementary Material 3

## Data Availability

No datasets were generated or analysed during the current study.
