# Supplementary figures and images for: Efficacy of mind maps and concept maps in enhancing academic performance among undergraduate medical students in the preclinical stage: a systematic review
Source: Adv Health Sci Educ Theory Pract. 2025 Jun 24;31(2):705–25. doi: 10.1007/s10459-025-10437-4 (PMC13046634; doi:10.1007/s10459-025-10437-4)

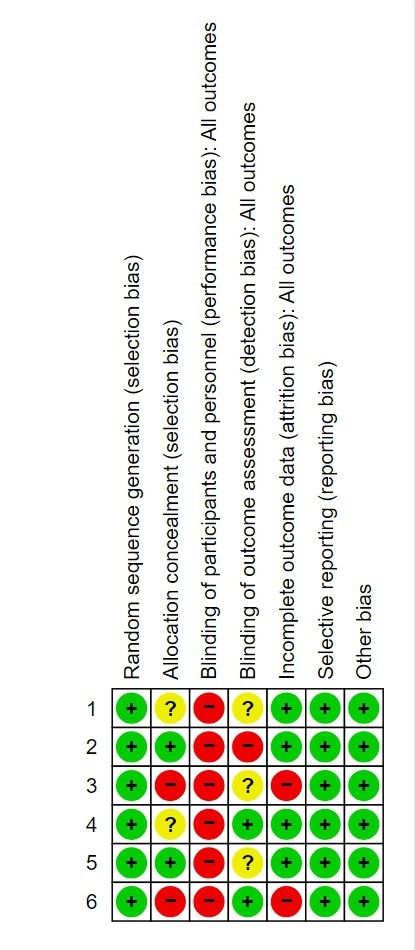

Supplement: Supplementary file 1 — Supplementary Material 1 [file 10459_2025_10437_MOESM1_ESM.jpeg]

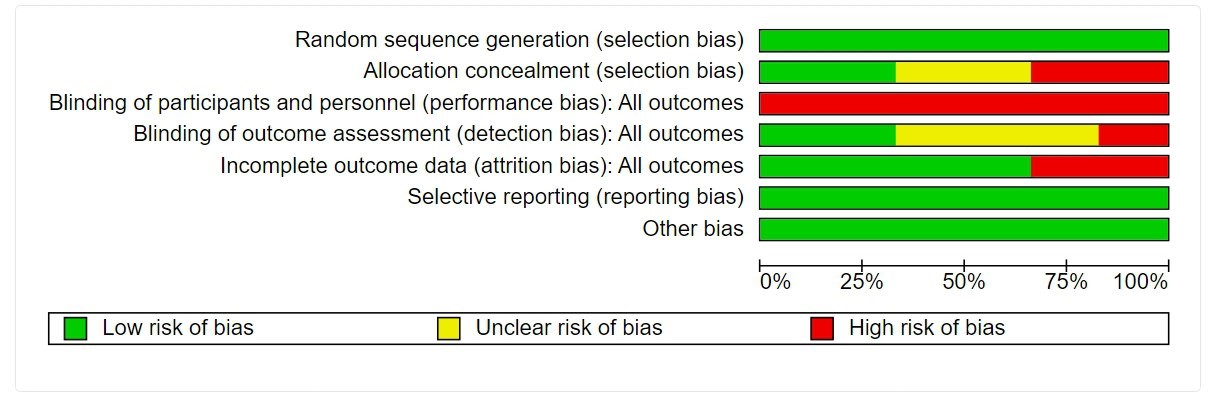

Supplement: Supplementary file 2 — Supplementary Material 2 [file 10459_2025_10437_MOESM2_ESM.jpeg]
