# Supplementary material for: Efficacy of mind maps and concept maps in enhancing academic performance among undergraduate medical students in the preclinical stage: a systematic review
Source: Adv Health Sci Educ Theory Pract. 2025 Jun 24;31(2):705–25. doi: 10.1007/s10459-025-10437-4 (PMC13046634; doi:10.1007/s10459-025-10437-4)
Supplement: Supplementary file 3 — Supplementary Material 3 [file 10459_2025_10437_MOESM3_ESM.docx]

**Database Search Strategy**

Table 1: Detailed search strategy

| Database | Search Strategy |
| --- | --- |
| PubMed | (("mind map*") OR ("concept map*")) AND ("medical student*") AND PUBYEAR > 2000 AND PUBYEAR < 2024 |
| Scopus | TITLE-ABS-KEY (((“mind map*”) OR (“concept map*”)) AND ("medical student*")) AND PUBYEAR > 2000 AND PUBYEAR < 2024 |
| Google Scholar | (("mind map*") OR ("concept map*")) AND ("medical student*") AND PUBYEAR > 2000 AND PUBYEAR < 2024 |
| Cochrane Library | (("mind map*") OR ("concept map*")) AND ("medical student*") AND PUBYEAR > 2000 AND PUBYEAR < 2024 |
